# Dynamic Frequency Analyses of Lower Extremity Muscles during Sit-To-Stand Motion for the Patients with Knee Osteoarthritis

**DOI:** 10.1371/journal.pone.0147496

**Published:** 2016-01-25

**Authors:** Masaya Anan, Koichi Shinkoda, Kentaro Suzuki, Masahide Yagi, Nobuhiro Kito

**Affiliations:** 1 Department of Biomechanics, Institute of Biomedical and Health Sciences, Hiroshima University, Hiroshima-shi, Hiroshima, Japan; 2 Center for Advanced Practice and Research of Rehabilitation, Hiroshima University, Hiroshima-shi, Hiroshima, Japan; 3 Nagoya University Hospital, Nagoya-shi, Nagoya, Japan; 4 Midorii Orthopaedics Joint Reconstruction & Arthroscopy, Hiroshima-shi, Hiroshima, Japan; 5 Hiroshima International University, Higashi-Hiroshima, Hiroshima, Japan; University of Umea, SWEDEN

## Abstract

**Objective:**

Muscle activities during the sit-to-stand motion (STS) are characterized by coordinated movements between hip extensors and knee extensors. However, previous reports regarding the STS and lower extremity muscle activities have focused on some quantitative assessment, but little qualitative research. This study aimed to examine the muscle activities of the lower extremity both quantitatively and qualitatively.

**Methods:**

Study participants included 13 patients with knee osteoarthritis (knee OA) and 11 age-matched asymptomatic controls. The task was STS from a chair with a height-adjustable seat. EMG activities were acquired using surface electromyogram. The root mean square signals normalized as a percentage of maximum voluntary isometric contraction values (RMS%MVC) and the mean power frequency (MPF) were calculated.

**Results:**

During STS, knee OA patients had increased RMS%MVC of the vastus medialis and raised MPF of the rectus femoris before buttocks-off.

**Conclusion:**

These findings suggest that STS of knee OA patients not only increased relative muscle activity of the vastus medialis, but also enlisted the rectus femoris in knee extension to improve muscle contraction force by activating more type II fibers to accomplish buttocks-off.

## Introduction

Knee osteoarthritis (knee OA) is a degenerative joint disease characterized by the accumulated mechanical stress leading to pathological changes, such as articular cartilage degeneration and failure, osteophytes formation [1]. Clinical presentation includes knee joint pain, crepitation with joint motion, and localized inflammation as well as functional disorders, such as knee excursion disturbance and quadriceps femoris muscle weakness [2]. In addition, there is an increase in tectonic problems, such as varus deformity of the knee [3] and knee instability [4], thereby impeding the ability to perform many motions used in daily life.

Sit-to-stand (STS) is a routine motion repeated several times every day. STS is a dynamic motion that requires extensive joint movement in the lower extremities and trunk, and also subjects the lower extremity joints to heavy loads that accompany the posture change from sitting to standing [5]. In our previous study, STS in patients with knee OA had reduced energy absorption in the knee extensors from the shank forward lean movement after buttocks-off, had reduced knee extensor efficiency, and used more physiological energy [6]. Previous studies of STS and lower extremity muscle activities indicate that knee extensors are more involved than any other major lower extremity muscle [7,8]. Muscular activity during STS is characterized by coordinated movement of hip extensors and knee extensors [9]. Likewise, it has been reported that knee OA patients have decreased quadriceps muscle force [2,10] and that muscle force is related to OA progression and knee instability [11]. However, only quantitative reports have been obtained regarding muscles during the STS of knee OA patients [12,13]. Qualitative assessment of muscles using muscle fiber composition ratios obtained from power spectral density [14–16] or prediction of muscle contraction patterns of motor units [17] have yet to be performed. Wavelet transform (WT), an analysis method developed for non-stationary signal waveforms with excellent temporal resolution has also been applied to EMG and can be used for qualitative muscle assessment [18]. An advantage of WT is that analytic signals do not degrade even with non-stationary brief EMG, allowing analysis of temporal data as well as fast Fourier transform (FFT) frequency data [18]. Kato and Jingushi [18] have reported frequency characteristics and histological features of the gluteus medius while walking in patients with hip osteoarthritis using WT. WT may demonstrate changes in frequency during dynamic contraction. As such, frequency power spectrum analysis is characterized by the ability to predict activity patterns of motor units that regulate the nervous system.

This study aimed to clarify quantitatively and qualitatively the characteristics of muscle activities in knee OA patients during STS. Elucidation of whether or not knee OA patients are performing STS efficiently by examining muscle activity patterns may allow us to propose innovative physical therapy methods for knee OA patients.

## Methods

### Subjects

Study participants included 13 women diagnosed with knee OA (knee OA group) and 11 women who did not fulfill the American College of Rheumatology clinical diagnostic criteria for knee OA [19] formed as age-matched asymptomatic controls (control group) for comparisons. Table 1 shows the age, body height, body mass, and body mass index (BMI) of study participants. The knee OA group experienced narrowing of knee joint space and formation of osteophytes. Knee OA patients with completely blocked knee joint space or with knee flexion contraction of 15° or more were excluded from the study. Other exclusion criteria included a history of central nervous system disease, lower extremity artificial joint replacement, lower extremity trauma or surgery, serious heart or lung disease, and rheumatoid arthritis. Patients using a cane in their daily lives or who had difficulty walking without a walking aid device were also excluded. Before and after X-rays photographs of the knee joint taken when participants were standing on both legs were used to determine the seriousness of knee OA with the Kellgren-Lawrence grading scale [20]. Eight participants had grade II OA, and five had grade III.

**Table 1:** Characteristics of the knee OA group and the control group.

	Knee OA group (n = 13)	Control group (n = 11)
Age [years]	71.1±4.7	68.9±4.2
Body height [m]	1.50±0.04	1.52±0.04
Body mass [kg]	54.3±6.3*	49.0±7.1
BMI [kg/m^2^]	24.3±3.2*	21.2±2.6
OA grade	II: 8, III: 5	

mean ± standard deviation,

*: p<0.05

Prior to the experiment, the purpose of this study was thoroughly explained to participants, and oral and written consent was obtained. This study was undertaken with the approval of the Ethics Committee of Division of Physical Therapy and Occupational Therapy Sciences, Graduate School of Health Sciences, Hiroshima University (No.0826).

### STS measurement

A chair with a height-adjustable seat without a backrest and armrests was used for measurements, with the ordinary seated position taken as the initial sitting posture. The vertical distance between the lateral knee joint space and the floor was adopted as the seat height. The shanks were maintained in a vertical position. Participants were barefoot and the feet width was set as the distance between the anterior superior iliac spines. Sitting depth was set so that the midpoints between the greater trochanter and lateral epicondyle of the femur were aligned to the front edge of the seat. Participants were instructed to face forward while sitting and to fold their arms on their chest to avoid masking the markers.

The task was STS from the initial sitting posture. Participants started the motion in their own time after the examiner’s oral cue and the motion velocity was based on participant comfort. Participants practiced the motion sufficiently prior to the motion being measured five times.

Infrared-reflecting markers were attached to four landmarks including a pair at the acromion, inferior edge of the last rib. These three-dimensional coordinates were collected at a sampling rate of 100 frame/s by Vicon MX, a three-dimensional motion analysis system (Vicon Motion Systems, Oxford, UK) with six infrared cameras. At the same time, the three-dimensional floor reaction forces were collected by four force plates (Tec Gihan, Uji, Japan) at a sampling frequency of 1,000 Hz. Initiation of the motion was defined as the last transition from negative to positive angular velocities before the occurrence of the maximum trunk forward lean angular velocity. Termination of the motion was defined as the first transition from negative to positive angular velocities after the occurrence of the minimum trunk forward lean angular velocity. Buttocks-off was defined as the instant the vertical vector of the floor reaction force of the buttocks was less than 10 N. Data from motion initiation to termination were normalized to 100%, and the buttocks-off time was defined as 50% time.

The EMG activities of four muscles were acquired using an EMG Master, surface electromyogram (Mediarea Support Business Union, Okayama, Japan) with a pass-band of 15–500 Hz at a sampling frequency of 1,000 Hz. The skin surface was prepared by shaving and cleaning with the skin pre-processing agent, Skin Pure (Nihon Kohden, Tokyo, Japan). Bipolar surface circular Ag/AgCl electrodes 34 mm in diameter, Blue-sensor M-00-S (Ambu, Ølstykke, Denmark) were placed 30 mm center-to-center in line with the muscle fibers over the gluteus maximus (GMA), medial hamstrings (MHam), vastus medialis (VM), and rectus femoris (RF). Electrodes were placed according to SENIAM guidelines [21]. The GMA electrode was placed at 50% on the line from the iliac crest to the greater trochanter. The MHam electrode was placed at 50% on the line from the sulcus glutealis to the knee joint. The VM electrode was placed 2 cm medial to the superior border of the patella with an inclination of 55°. The RF electrode was placed at 50% on the line from the anterior superior iliac spine to the knee joint. A reference electrode was placed over the radial styloid process. The left limb was tested in the control group, while the affected limb (or the most symptomatic in the eight participants who reported bilateral symptoms) was tested in the knee OA group.

Three maximum voluntary isometric contractions (MVC) for 3 seconds of each muscle were measured at the position of Normal level on the basis of manual muscle testing [22]. The highest root mean square (RMS) signals averaged over 50 ms intervals were calculated. RMS signals during STS were calculated with a step of 5% samples and normalized as a percentage of MVC values (RMS%MVC).

Frequency power spectrum analysis breaks down EMG interference waveforms into waves of various frequencies and arranges them in order of frequency, with the horizontal axis representing frequency and the vertical axis representing frequency power density. Solomonow et al. [23] reported that an increase in the average conduction velocity during motor unit recruitment greatly contributes to variations in electromyogram median frequency. On the muscle fiber level, slow-twitch fibers (type I fibers) are thought to be represented by low-frequency band components and fast-twitch fibers (type II fibers) by high-frequency band components [24,25]. These power spectral density data make it possible to estimate muscle fatigue, muscle fiber composition ratio, and motor unit activity patterns. The mean power frequency (MPF) during STS was acquired using the Km-Mercury, EMG monitoring program (Mediarea Support Business Union, Okayama, Japan). Raw data spectra were transformed using continuous WT with a Gabor mother wavelet for the frequency range 15–500 Hz. Each spectrum was calculated from a signal period of 5% duration and consisted of 25 points. In addition, frequency bands were divided into three groups: lower frequency band (0–45 Hz), middle frequency band (46–80 Hz), and higher frequency band (81–500 Hz) [26]. The proportion of lower and higher frequency bands in relation to a whole frequency band area was calculated for each muscle. The mean of the data collected in five STSs is represented.

### Testing of maximum hip and knee extension force

Maximum isometric hip and knee extensor force were tested using a hand-held dynamometer (HHD), μTas MT-1 (Anima, Chofu, Japan). The initial position of the knee extensor was sitting, with shanks vertical and feet not touching the floor. The test position was with the knee at 60° flexion. The sensor attachment of the HHD was always held perpendicular to the front shank just proximal to the ankle, and it was fixed by the belt to maintain the test position. The initial test position of the hip extensor was prone, and the test position was with the hip extended 10° and the knee straight. The sensor attachment of the HHD was always held perpendicular to the posterior aspect of the thigh proximal to the knee joint, and participants were fixed by the belt to maintain the test position.

Prticipants maintained the maximum exertion for 5 seconds, and then the maximum force was recorded. Maximum exertion was tested three times, separated by rest periods of approximately 30 seconds. The mean of the data tested three times is represented. To eliminate the influence of subject body mass, the value was normalized to the subject’s body mass.

### Statistical analyses

The data were expressed as mean ± standard deviation. Statistical analyses were executed using statistical software SPSS ver. 17.0 J for Windows (SPSS Japan, Tokyo, Japan). It has been reported that muscle force decreases with aging [27]. Consequently, analyses of covariance incorporated age as a covariant for comparing muscle force between the knee OA and control groups. Two-sample t-test was used for comparing between the knee OA and control groups in the case of homoscedasticity: the Welch test was used in the case of heteroscedasticity. The significant level was set at less than 5%.

## Results

Knee extensor forces in the knee OA group were significantly smaller than those in the control group (0.83±0.25 N·m/kg vs. 1.16±0.21 N·m/kg; p<0.05), but hip extensor forces showed no significant differences between the two groups (0.68±0.33 N·m/kg vs. 0.81±0.25 N·m/kg)(S2 Table).

**Fig 1:**
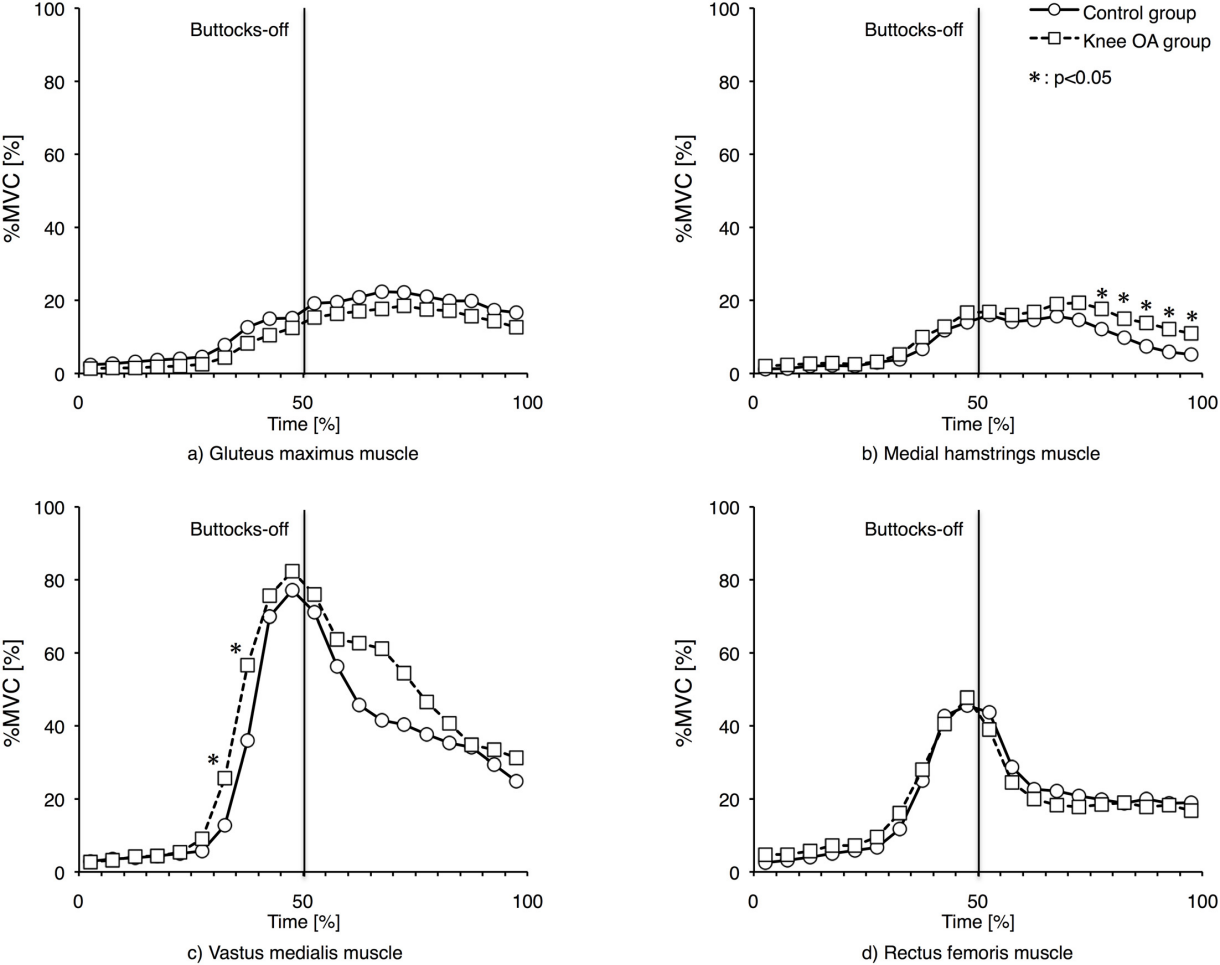
RMS%MVC changes of each muscle. a) Gluteus maximus, b) Medial hamstrings, c) Vastus medialis, d) Rectus femoris RMS%MVC changes of gluteus maximus, medial hamstrings, vastus medialis, and rectus femoris during STS duration.

Fig 1 shows changes in RMS%MVC of each muscle. RMS%MVCs of GMA and RF showed no significant differences between the two groups. RMS%MVCs of MHam for 75–80%, 80–85%, 85–90%, 90–95%, and 95–100% were significantly larger in knee OA group than in the control group (p<0.05). RMS%MVCs of VM for 30–35% and 35–40% were significantly larger in knee OA group than in the control group (p<0.05).

**Fig 2:**
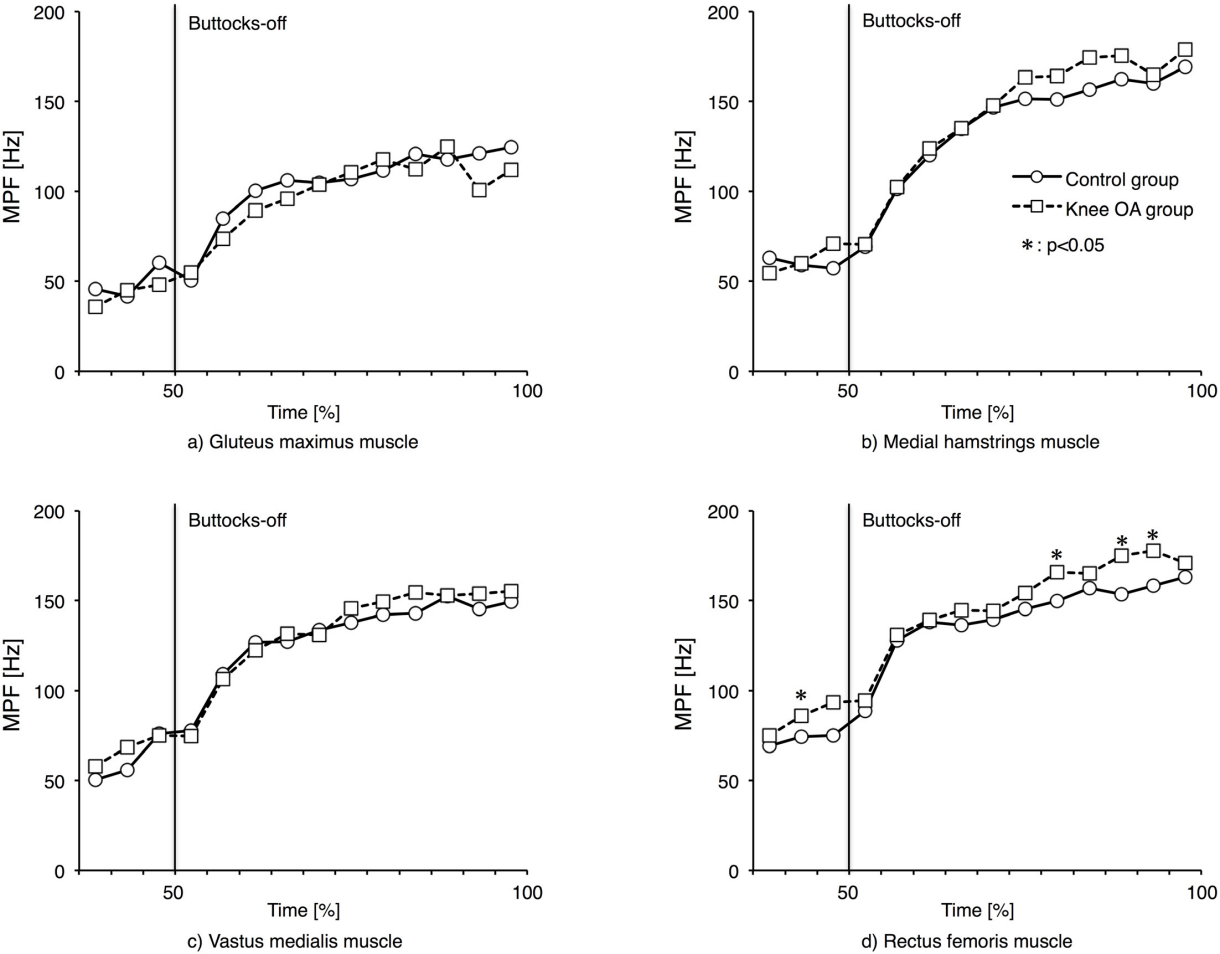
Mean power frequency changes of each muscle. a) Gluteus maximus, b) Medial hamstrings, c) Vastus medialis, d) Rectus femoris. Mean power frequency changes of gluteus maximus, medial hamstrings, vastus medialis, and rectus femoris during STS duration.

Fig 2 shows changes in MPF of each muscle. MPFs of GMA, MHam, and VM showed no significant differences between the two groups. MPFs of RF for 40–45%, 75–80%, 85–90%, and 90–95% were significantly larger in knee OA group than in the control group (p<0.05). Table 2 shows the proportion of each frequency band for RF that MPF showed significant difference between the two groups. As a result, the lower frequency band for 35–40%, 40–45%, and 45–50% was smaller in knee OA group than in the control group (p<0.05). In contrast, the higher frequency band for 35–40% and 40–45% was larger in knee OA group than in control group (p<0.05).

**Table 2:** The rate of each frequency band for rectus femoris.

		Sitting phase (%)				Standing phase (%)								
		35–40	40–45	45–50	50–55	55–60	60–65	65–70	70–75	75–80	80–85	85–90	90–95	95–100
**Lower frequency band**														
Knee OA group	Mean	56.8	55.5	52.2	41.1	33.6	28.8	29.8	26.2	23.2	22.2	20.8	21.0	22.8
	SD	11.0	9.2	11.0	10.2	9.0	8.1	9.7	8.3	6.1	5.9	7.4	7.0	6.5
Control group	Mean	67.2	63.8	62.0	44.0	35.1	35.3	29.6	31.0	26.6	23.9	26.8	23.2	23.8
	SD	10.5	9.1	8.8	5.8	8.4	8.4	5.0	7.6	6.0	7.9	7.9	5.9	6.2
	P-value	**0.03**	**0.04**	**0.03**	0.41	0.68	0.07	0.95	0.15	0.18	0.55	0.07	0.42	0.71
**Higher frequency band**														
Knee OA group	Mean	22.8	24.9	28.5	39.0	45.4	49.3	51.7	52.8	59.2	59.6	62.3	64.1	59.2
	SD	9.1	9.2	11.3	10.8	12.2	10.0	12.1	8.8	10.4	9.5	10.1	8.3	8.0
Control group	Mean	15.6	18.1	21.8	36.6	44.7	42.4	46.2	50.1	52.9	56.5	55.1	57.5	59.1
	SD	6.2	4.7	7.1	4.8	10.4	11.5	7.4	8.4	7.9	7.6	9.0	8.0	8.5
	P-value	**0.04**	**0.04**	0.10	0.50	0.87	0.13	0.20	0.44	0.11	0.40	0.08	0.06	0.96

The proportion of lower and higher frequency bands in relation to a whole frequency band area for RF that MPF showed significant differences (bold letters) between the two groups. Upper part shows lower frequency band (0–45 Hz) and lower part shows higher frequency band (81–500 Hz).

## Discussion

This study aimed to clarify quantitatively and qualitatively the characteristics of muscle activities in knee OA patients during STS. We have demonstrated that STS of knee OA patients not only increased relative muscle activity of the VM, but also enlisted the RF in knee extension to improve muscle contraction force by activating more type II fibers to accomplish buttocks-off.

STS is a complicated motion to perform and has two components: (1) transition from a wide base of support (BOS) created by the buttocks, thighs and feet to a narrow BOS created only by the feet and (2) lifting the center of mass (COM) from the height of sitting to that of standing [28,29]. Millington et al. [30] have reported that in STS, the VM and RF become active before knee extension begins, but the MHam and GMA become active after the movement begins. The current study showed the same trend. In addition, the RMS%MVC of VM and RF peaked during the buttocks-off. Previous reports regarding STS and lower extremity muscle activities found that knee extensors are more involved than any other major lower extremity muscle [7,8]. This suggests that the RF and VM are the principal muscles used to accomplish buttocks-off. However, it has been reported that knee OA patients have decreased quadriceps muscle force [2] and decreased joint proprioception [31], preventing them from effectively using their knee extensors [32]. In this study, RMS%MVCs of VM for 30–35% and 35–40% were significantly larger in the knee OA group than in the control group. The RMS value is generally used as an index for muscle activity because it has a direct relationship with muscle tension during isometric contraction [33]. Knee joint maximum extensor forces were significantly smaller in the knee OA group than in the control group. Moreover, body mass in the knee OA group was significantly heavier than that in the control group. This result may reflect a relative increase in muscle activity due to a decrease in the absolute force of the knee extensors and the heavier mass to lift in the knee OA group, suggesting that more activation of VM was necessary to accomplish buttocks-off.

The amplitude of GMA and MHam activity peaked during lower extremity extension movement after buttocks-off, suggesting that these muscles perform hip extension and thus participate with knee extensors in the upward movement of the COM after buttocks-off. Although hamstrings are generally active in knee flexion, they are also active in knee extension via co-contraction with the RF in closed kinetic chain (CKC) such as in STS. Co-contraction during CKC is thought to stabilize and protect the knee joint [34]. Patsika et al. [12] have reported that MHam, an antagonistic muscle, exhibits increased activity during STS in patients with knee OA. This study achieved the same results, with the RMS%MVCs of MHam for 75–80%, 80–85%, 85–90%, 90–95%, and 95–100% in the latter half of lower extremity extension being significantly larger in the knee OA group than in the control group. Hamstrings act as extensors during a CKC when the knee joint is at a flexion position of 0–60° [35]. This suggests that the knee OA group preferentially used MHam to extend lower extremity joints.

A frequency power spectrum is interfering waveforms in EMG broken down into waves by frequencies and arranged in order with the horizontal axis representing frequency and the vertical axis representing frequency power density. This quantifies the activity status of motor units with MPF as the mean value of their frequencies. In EMG frequency analysis, low-frequency band reflect increased activity of type I fibers, that are controlled by tonic motor units, while high-frequency bands reflect increased activity of type II fibers, that are controlled by phasic motor units [24]. A concept known as the size principle suggests that muscle contraction begins with type I fibers, that have weak-twitch force, and type II fibers are mobilized as the contractile rate increases [36]. This study showed a shift toward high frequency in the power spectrum and increased after buttocks-off in all muscles tested for both groups. This appears to be similar to the shift in power spectrum observed during the initial stance phase of walking in the study conducted by Kato and Jingushi [18]. It seems that, since buttocks-off narrows the BOS and lifts the COM from sitting height to standing height, the number of participating motor units and the number of mobilized fast-twitch fibers increase in conjunction, resulting in increased MPF.

In this study, MPFs of RF for 40–45%, 75–80%, 85–90%, and 90–95% were significantly larger in the knee OA group than those in the control group. The low frequency bands were smaller while the high frequency bands were larger for 35–40%, 40–45%, and 45–50% in the knee OA group compare the control group. Quadriceps muscles contain many type II fibers that are preferential in instantaneous movements [37] and are thought to influence moment power exertion [38]. Deterioration of quadriceps muscle force manifests itself in fast-twitch fibers [39], and muscle atrophy occurs more often in the VM than in the RF [40]. We did not analyze MPF during MVC, but muscle fiber conduction velocity has been reported to be significantly slower due to weakness of the quadriceps muscle [41], and a decrease in MPF indicates a slowing of conduction velocity [42]. Therefore, it is possible that muscle fiber conduction velocity during MVC in the knee OA group may be slower and MPF during MVC may have decreased. These data suggest that to accomplish the buttocks-off, which is mechanically demanding, the knee OA group not only increased relative muscle activity of the VM, but also enlisted the RF in knee extension to improve muscle contraction force by more activating type II fibers. Kumamoto et al. [43] stated that mono-articular muscles are for external output and used for support against gravity, and that bi-articular muscles are used for controlling direction of external output through co-contraction with mono-articular muscles. Bi-articular muscles also contribute to effective transmission of muscle contraction power, to achieve what is known as the power transfer effect [44]. It has also been reported that the RF exerts tensile force in STS as well, acting like a tendon, and converts muscle force of hip extensors into knee extensor force [45]. All this suggests that knee OA patients may have altered muscle contraction patterns that may have caused their decreased power transfer effects.

Our results indicate that STS of knee OA patients not only increases relative muscle activity of the VM, but also enlists the RF in knee extension, thereby improving muscle contraction force via activation of more type II fibers to accomplish buttocks-off. In practice, acquiring efficiency in appropriate exertion using hip and knee extensors may decrease overloading of knee extensors, which may prevent qualitative variations of RF.

A limitation of this study is that as a result of this study’s small number of subjects, the statistical power of the motion analysis findings is low. Furthermore, we did not consider the effect of pain. It has been reported that quadriceps weakness is related to pain [45]. Further studies are to clarify that muscle activity in knee OA patients vary qualitatively under the influence of the pain. And then, an increase in MPF does not necessarily indicate that more fast-twitch motor units are active, as a higher firing rate of slow-twitch motor units is also possible [42]. So, we must investigate whether the cause of MPF increasing of RF during STS in patients with knee OA is related to muscle or motion characteristics. This enables us to know the impairment factors lead to the pathologies and progressions and to reach a promising physical therapy for knee OA.

## Conclusions

STS of knee OA patients not only increased relative muscle activity of the VM, but also enlisted the RF in knee extension to improve muscle contraction force by activating more type II fibers to accomplish buttocks-off. This may indicate that STS accomplishment itself was maintained, while muscle activity and contraction forms required for STS varied. Thus, it appears that acquiring efficiency of appropriate exertion using hip and knee extensors decrease overloading of knee extensors. Our findings provide new insights into quantitative and qualitative improvement of muscle function with knee OA patients.

## Supporting Information

S1 Table The detailed data of characteristics of the knee OA group and the control group. (PDF)

S2 Table The detailed data of extensor force of the knee OA group and the control group. (PDF)

S3 Table The detailed data of RMS%MVC changes of each muscle of the knee OA group and the control group. (PDF)

S4 Table The detailed data of mean power frequency changes of each muscle of the knee OA group and the control group. (PDF)

S5 Table The detailed data of the rate of each frequency band for rectus femoris of the knee OA group and the control group. (PDF)
