# Supplementary material for: Dynamic Frequency Analyses of Lower Extremity Muscles during Sit-To-Stand Motion for the Patients with Knee Osteoarthritis
Source: PLoS One. 2016 Jan 25;11(1):e0147496. doi: 10.1371/journal.pone.0147496 (PMC4726819; doi:10.1371/journal.pone.0147496)
Supplement: S1 Table — (PDF) [file pone.0147496.s001.pdf]

S1 Table. The detailed data of characteristics of the knee OA group and the control group.

Knee OA Group	Age [years]	Body height [m]	Body mass [kg]	BMI [kg/m2]	OA grade
OA001	69	1.47	52.0	24.0	III
OA002	66	1.48	58.0	26.3	II
OA003	80	1.57	56.0	22.9	III
OA004	68	1.47	59.5	27.6	II
OA005	70	1.49	52.5	23.8	II
OA006	70	1.46	60.0	28.0	III
OA007	61	1.57	60.0	24.4	II
OA008	74	1.46	62.0	29.3	II
OA009	73	1.44	52.5	25.3	II
OA010	76	1.47	50.5	23.5	II
OA011	75	1.58	50.5	20.3	II
OA012	69	1.52	55.5	24.1	III
OA013	73	1.48	37.0	16.8	III
Mean	71.1	1.50	54.3	24.3	
Standard deviation	4.7	0.04	6.3	3.2	

Control group	Age [years]	Body height [m]	Body mass [kg]	BMI [kg/m2]
Cont001	76	1.50	55.0	24.3
Cont002	70	1.55	44.5	18.6
Cont003	65	1.53	55.0	23.6
Cont004	70	1.60	59.5	23.2
Cont005	66	1.46	36.0	16.8
Cont006	66	1.48	48.0	21.9
Cont007	69	1.56	52.0	21.3
Cont008	67	1.51	42.0	18.4
Cont009	62	1.50	51.5	22.8
Cont010	71	1.48	40.0	18.3
Cont011	76	1.52	55.5	24.0
Mean	68.9	1.52	49.0	21.2
Standard deviation	4.2	0.04	7.1	2.6
