# Supplementary material for: Dynamic Frequency Analyses of Lower Extremity Muscles during Sit-To-Stand Motion for the Patients with Knee Osteoarthritis
Source: PLoS One. 2016 Jan 25;11(1):e0147496. doi: 10.1371/journal.pone.0147496 (PMC4726819; doi:10.1371/journal.pone.0147496)
Supplement: S2 Table — (PDF) [file pone.0147496.s002.pdf]

S2 Table. The detailed data of extensor force of the knee OA group and the control group.

Knee OA Group	Hip extensor force [N･m/kg]	Knee extensor force [N･m/kg]
OA001	0.53	0.75
OA002	0.40	0.53
OA003	0.74	1.30
OA004	0.10	0.78
OA005	0.30	0.65
OA006	0.95	0.84
OA007	0.59	0.77
OA008	1.27	1.07
OA009	0.49	0.57
OA010	0.67	0.75
OA011	1.22	0.93
OA012	0.59	0.59
OA013	0.98	1.31
Mean	0.68	0.83
Standard deviation	0.33	0.25

Control group	Hip extensor force [N･m/kg]	Knee extensor force [N･m/kg]
Cont001	0.41	1.42
Cont002	0.53	1.09
Cont003	1.00	1.06
Cont004	0.86	1.22
Cont005	1.24	1.27
Cont006	1.06	1.31
Cont007	0.74	1.36
Cont008	0.45	1.16
Cont009	0.91	1.18
Cont010	0.90	1.04
Cont011	0.81	0.62
Mean	0.81	1.16
Standard deviation	0.25	0.21
