# Supplementary material for: Dynamic Frequency Analyses of Lower Extremity Muscles during Sit-To-Stand Motion for the Patients with Knee Osteoarthritis
Source: PLoS One. 2016 Jan 25;11(1):e0147496. doi: 10.1371/journal.pone.0147496 (PMC4726819; doi:10.1371/journal.pone.0147496)
Supplement: S3 Table — (PDF) [file pone.0147496.s003.pdf]

S3 Table. The detailed data of RMS%MVC changes of each muscle of the knee OA group and the control group.

a) Gluteus maximus

Knee OA Group	0～5%	5～10%	10～15%	15～20%	20～25%	25～30%	30～35%	35～40%	40～45%	45～50%	50～55%	55～60%	60～65%	65～70%	70～75%	75～80%	80～85%	85～90%	90～95%	95～100%
OA001	0.005882	0.006613	0.006442	0.008521	0.007364	0.011209	0.020686	0.046171	0.082276	0.095607	0.151049	0.136492	0.179998	0.184881	0.18721	0.160077	0.169309	0.124454	0.115218	0.114512
OA002	0.010089	0.013854	0.017855	0.023034	0.021894	0.030762	0.031363	0.04321	0.062063	0.103058	0.122911	0.144602	0.144191	0.192015	0.183249	0.170871	0.169257	0.146337	0.153339	0.133981
OA003	0.0113	0.011581	0.012824	0.013625	0.013296	0.016834	0.022265	0.062324	0.148724	0.160805	0.183677	0.17475	0.180043	0.213833	0.205522	0.143694	0.10915	0.114782	0.097364	0.088473
OA004	0.007875	0.009231	0.008331	0.011165	0.013484	0.019576	0.036401	0.06645	0.071694	0.071602	0.108135	0.117341	0.135311	0.111563	0.103102	0.074213	0.057176	0.037366	0.016452	0.022451
OA005	0.00482	0.004677	0.005512	0.006262	0.006971	0.007315	0.012457	0.022711	0.036044	0.047508	0.065299	0.070138	0.090078	0.088623	0.106339	0.127168	0.152004	0.162022	0.183528	0.152385
OA006	0.005738	0.006032	0.007339	0.007044	0.005772	0.006761	0.009715	0.025617	0.044721	0.060958	0.057867	0.068228	0.086996	0.088728	0.090065	0.070265	0.039956	0.020229	0.016005	0.014803
OA007	0.04615	0.048345	0.047863	0.049428	0.053754	0.049978	0.093961	0.147619	0.155631	0.177056	0.189265	0.227724	0.205742	0.237548	0.239497	0.205937	0.196241	0.156811	0.105728	0.101119
OA008	0.014234	0.016175	0.016525	0.019577	0.019957	0.020904	0.024263	0.037558	0.052502	0.055473	0.069443	0.086725	0.08717	0.087837	0.137973	0.147396	0.172252	0.175504	0.160734	0.154895
OA009	0.009982	0.009529	0.01175	0.013092	0.013659	0.015962	0.02971	0.11952	0.109036	0.111841	0.139949	0.173462	0.182272	0.142758	0.127511	0.0978	0.066947	0.033883	0.034564	0.037577
OA010	0.022236	0.026133	0.030124	0.057399	0.055699	0.063361	0.110452	0.167894	0.153481	0.27472	0.21836	0.242084	0.276115	0.260892	0.267573	0.297747	0.411641	0.415165	0.347913	0.239807
OA011	0.010168	0.009406	0.009243	0.012629	0.016503	0.039214	0.085147	0.172896	0.227453	0.134048	0.288919	0.257598	0.247651	0.321688	0.346874	0.353347	0.31318	0.30629	0.32039	0.26205
OA012	0.015353	0.016719	0.016483	0.016873	0.025351	0.033275	0.053641	0.099707	0.122754	0.128769	0.185183	0.195445	0.19939	0.206404	0.259529	0.293898	0.285608	0.270901	0.247631	0.27471
OA013	0.007318	0.010097	0.009793	0.009606	0.013044	0.018166	0.037004	0.053391	0.096611	0.193566	0.215829	0.227862	0.186299	0.165883	0.148576	0.126842	0.092421	0.075906	0.056166	0.044313
Mean	0.013165	0.014492	0.015391	0.019097	0.020519	0.02564	0.04362	0.081928	0.104845	0.124232	0.15353	0.163265	0.169327	0.177127	0.184848	0.174558	0.171934	0.156896	0.142695	0.126237
Standard deviation	0.010555	0.011183	0.011305	0.015418	0.015597	0.01634	0.031308	0.051569	0.053231	0.062332	0.066099	0.062909	0.056874	0.069768	0.073759	0.085773	0.104957	0.110935	0.104569	0.085444

Control group	0～5%	5～10%	10～15%	15～20%	20～25%	25～30%	30～35%	35～40%	40～45%	45～50%	50～55%	55～60%	60～65%	65～70%	70～75%	75～80%	80～85%	85～90%	90～95%	95～100%
Cont001	0.119065	0.13008	0.151198	0.192116	0.207902	0.203757	0.362449	0.441156	0.317431	0.263197	0.262454	0.254399	0.279412	0.319656	0.363344	0.350716	0.324521	0.370023	0.358823	0.39471
Cont002	0.007191	0.00755	0.008481	0.008495	0.008599	0.010001	0.015756	0.054451	0.123688	0.103404	0.133465	0.153677	0.151545	0.149456	0.153239	0.152989	0.115781	0.122669	0.106224	0.130174
Cont003	0.014457	0.017487	0.015771	0.012023	0.016308	0.020645	0.037516	0.053809	0.070341	0.098131	0.096758	0.144739	0.194446	0.217402	0.19257	0.136932	0.097722	0.115525	0.100262	0.109216
Cont004	0.008135	0.008696	0.010873	0.014967	0.016418	0.02446	0.028069	0.047997	0.038645	0.031468	0.07388	0.089295	0.11153	0.143504	0.170905	0.176508	0.130597	0.104313	0.061497	0.037464
Cont005	0.003665	0.004088	0.00475	0.005945	0.010887	0.026198	0.052916	0.066195	0.076931	0.080099	0.071139	0.079612	0.108432	0.105105	0.096715	0.103756	0.077866	0.066651	0.05488	0.040811
Cont006	0.006593	0.00781	0.011413	0.011553	0.015196	0.026639	0.044779	0.097583	0.113463	0.097593	0.085277	0.090954	0.098009	0.100352	0.107157	0.124156	0.13823	0.16318	0.116543	0.106845
Cont007	0.024552	0.028563	0.039657	0.045156	0.054016	0.053166	0.057818	0.096835	0.131205	0.219329	0.515688	0.556895	0.590332	0.578284	0.374225	0.233203	0.208081	0.11848	0.124404	0.107064
Cont008	0.032939	0.047785	0.04703	0.053696	0.050495	0.060884	0.076586	0.137421	0.205141	0.20429	0.208067	0.17224	0.195575	0.240363	0.40528	0.608614	0.837971	0.946495	0.835184	0.771951
Cont009	0.018347	0.017092	0.019803	0.020794	0.022783	0.026809	0.031538	0.078652	0.191377	0.273141	0.317353	0.255313	0.201023	0.217294	0.162563	0.091851	0.064108	0.051994	0.043149	0.039258
Cont010	0.007541	0.006666	0.008717	0.008599	0.007948	0.015929	0.078085	0.165175	0.210903	0.17131	0.18517	0.198947	0.219593	0.248591	0.256246	0.190736	0.073427	0.023242	0.012555	0.011829
Cont011	0.024403	0.026947	0.02983	0.029833	0.033565	0.030321	0.071733	0.14509	0.163072	0.130251	0.155491	0.150687	0.13658	0.141912	0.16269	0.143374	0.109694	0.093459	0.098376	0.078245
Mean	0.024263	0.027524	0.031593	0.036652	0.040374	0.045346	0.077931	0.125851	0.149291	0.152019	0.19134	0.19516	0.207862	0.223811	0.222267	0.210258	0.198	0.197821	0.173809	0.166142
Standard deviation	0.031278	0.034727	0.040018	0.051398	0.055126	0.052087	0.092076	0.106705	0.075754	0.075723	0.127754	0.127984	0.131911	0.129273	0.105337	0.143454	0.214535	0.252022	0.226207	0.215468

b) Medial hamstrings

Knee OA Group	0～5%	5～10%	10～15%	15～20%	20～25%	25～30%	30～35%	35～40%	40～45%	45～50%	50～55%	55～60%	60～65%	65～70%	70～75%	75～80%	80～85%	85～90%	90～95%	95～100%
OA001	0.005958	0.005966	0.007059	0.007987	0.008613	0.014913	0.015233	0.07346	0.082918	0.085028	0.078615	0.091278	0.109383	0.164409	0.203847	0.20644	0.226386	0.214127	0.137664	0.165523
OA002	0.03212	0.037446	0.043779	0.040967	0.041784	0.046088	0.07533	0.086416	0.093003	0.066884	0.068776	0.098351	0.119211	0.126887	0.086896	0.093724	0.082391	0.078036	0.074816	0.063789
OA003	0.006665	0.008799	0.00814	0.007743	0.008851	0.012328	0.018558	0.049685	0.136848	0.190723	0.170523	0.154338	0.174789	0.198824	0.170428	0.131873	0.124692	0.15818	0.132289	0.123797
OA004	0.006303	0.00754	0.00846	0.009662	0.015154	0.027735	0.037141	0.06235	0.117834	0.14329	0.183908	0.164599	0.131222	0.155293	0.153461	0.155526	0.106668	0.071693	0.044059	0.033348
OA005	0.009964	0.012326	0.014919	0.021709	0.022921	0.026991	0.047663	0.105739	0.21664	0.252322	0.217109	0.16939	0.191785	0.233872	0.264617	0.237941	0.221964	0.198902	0.210469	0.158708
OA006	0.013401	0.031531	0.036524	0.046227	0.032626	0.018569	0.029804	0.056825	0.095729	0.323028	0.305385	0.196164	0.232476	0.237109	0.213068	0.201144	0.16462	0.128022	0.089176	0.08766
OA007	0.009135	0.009626	0.010023	0.008742	0.010427	0.011861	0.026543	0.13927	0.179326	0.208496	0.171352	0.183709	0.195617	0.192648	0.180743	0.126808	0.056187	0.046016	0.042641	0.041232
OA008	0.041557	0.051871	0.059263	0.050426	0.030477	0.047961	0.102805	0.171988	0.174355	0.181083	0.208457	0.187441	0.144177	0.203879	0.251616	0.202459	0.191591	0.176909	0.13556	0.14228
OA009	0.00761	0.009189	0.007858	0.009045	0.00819	0.009737	0.020204	0.122585	0.17866	0.279627	0.302955	0.284646	0.304785	0.341167	0.332357	0.275527	0.254744	0.22236	0.221705	0.20361
OA010	0.062496	0.075393	0.078983	0.089801	0.078033	0.091095	0.089046	0.094302	0.070322	0.11292	0.124277	0.141184	0.113096	0.126189	0.152055	0.157231	0.133181	0.138619	0.12441	0.117121
OA011	0.008577	0.008668	0.0108	0.011953	0.018568	0.024974	0.039294	0.059744	0.085042	0.087298	0.141815	0.220684	0.215538	0.235977	0.23337	0.240662	0.171332	0.164397	0.17501	0.108824
OA012	0.049225	0.043966	0.049666	0.039447	0.039631	0.053483	0.081525	0.124841	0.107276	0.132296	0.131	0.132771	0.151393	0.15763	0.177339	0.174263	0.148927	0.135227	0.125561	0.123294
OA013	0.010578	0.00697	0.017503	0.018077	0.016374	0.024731	0.088908	0.146586	0.112276	0.093824	0.071977	0.06019	0.098666	0.098836	0.085915	0.082845	0.069851	0.060252	0.059896	0.05168
Mean	0.020276	0.023792	0.027152	0.02783	0.025511	0.031574	0.051696	0.099522	0.126941	0.165909	0.167396	0.160365	0.167857	0.190209	0.192747	0.17588	0.150195	0.137903	0.12102	0.109297
Standard deviation	0.01854	0.021426	0.023003	0.023577	0.018881	0.022033	0.030068	0.037629	0.044308	0.078406	0.074639	0.056543	0.057066	0.06118	0.066219	0.056002	0.060116	0.056734	0.05592	0.049856

Control group	0～5%	5～10%	10～15%	15～20%	20～25%	25～30%	30～35%	35～40%	40～45%	45～50%	50～55%	55～60%	60～65%	65～70%	70～75%	75～80%	80～85%	85～90%	90～95%	95～100%
Cont001	0.018114	0.018669	0.031286	0.03482	0.023483	0.026182	0.037376	0.09634	0.12192	0.207022	0.153602	0.102505	0.116837	0.147743	0.174291	0.153071	0.151164	0.12561	0.117133	0.11463
Cont002	0.007093	0.019713	0.041519	0.04362	0.036928	0.05749	0.040723	0.077901	0.076641	0.065017	0.120144	0.128021	0.112091	0.09622	0.092511	0.061383	0.0447	0.038243	0.038218	0.037646
Cont003	0.006794	0.012487	0.021023	0.023222	0.011368	0.015535	0.024917	0.042418	0.07492	0.118291	0.136491	0.124165	0.137874	0.16748	0.141427	0.094726	0.083597	0.054436	0.038107	0.033887
Cont004	0.014403	0.008151	0.01236	0.015668	0.029675	0.098854	0.134845	0.06113	0.066561	0.057059	0.088312	0.084802	0.097016	0.096523	0.090779	0.069733	0.039391	0.020236	0.01347	0.012719
Cont005	0.025312	0.022264	0.021525	0.018094	0.016833	0.02381	0.033049	0.098416	0.186068	0.12854	0.091194	0.106708	0.120933	0.189371	0.217386	0.218662	0.207204	0.179838	0.144112	0.119536
Cont006	0.010116	0.014104	0.017203	0.011377	0.013231	0.017384	0.05228	0.160467	0.253547	0.18792	0.225484	0.139784	0.119739	0.094796	0.065411	0.039259	0.036126	0.03895	0.025285	0.026309
Cont007	0.004726	0.005006	0.005949	0.00644	0.006232	0.005865	0.006194	0.045314	0.14974	0.247227	0.293091	0.28051	0.267966	0.252017	0.178697	0.112135	0.090992	0.05176	0.040264	0.043948
Cont008	0.024964	0.02901	0.031401	0.03201	0.039435	0.028567	0.02466	0.026725	0.074285	0.092041	0.134999	0.126872	0.14738	0.196745	0.238593	0.235615	0.167615	0.093544	0.060922	0.0532
Cont009	0.008315	0.00797	0.008523	0.010748	0.011735	0.014379	0.014799	0.067706	0.115742	0.16776	0.157091	0.137486	0.151953	0.184959	0.158557	0.137119	0.084861	0.061841	0.048122	0.048986
Cont010	0.008428	0.007096	0.007142	0.014903	0.010795	0.013431	0.02756	0.034163	0.059611	0.150782	0.135403	0.133536	0.112983	0.095312	0.077627	0.07271	0.064357	0.060485	0.051425	0.034292
Cont011	0.008258	0.010321	0.017016	0.025323	0.028425	0.026869	0.033539	0.068332	0.106809	0.11984	0.225034	0.197455	0.226321	0.192169	0.169127	0.142798	0.093744	0.081851	0.077243	0.056995
Mean	0.012411	0.014072	0.019541	0.021475	0.02074	0.029851	0.039086	0.07081	0.116895	0.140136	0.160077	0.141986	0.146463	0.155758	0.145855	0.121564	0.096705	0.073345	0.059482	0.052922
Standard deviation	0.006971	0.007154	0.010834	0.010991	0.010904	0.025281	0.032516	0.036124	0.056952	0.056394	0.059943	0.051502	0.050665	0.051238	0.055047	0.060603	0.053311	0.043599	0.037613	0.032509

c) Vastus medialis

Knee OA Group	0～5%	5～10%	10～15%	15～20%	20～25%	25～30%	30～35%	35～40%	40～45%	45～50%	50～55%	55～60%	60～65%	65～70%	70～75%	75～80%	80～85%	85～90%	90～95%	95～100%
OA001	0.010538	0.009973	0.009659	0.018058	0.044749	0.056013	0.160629	0.276488	0.39151	0.459883	0.653471	0.65171	0.682736	0.509784	0.456829	0.257407	0.148373	0.11229	0.123078	0.106922
OA002	0.068458	0.082024	0.086244	0.089851	0.104094	0.186068	0.662375	1.166321	1.256158	1.266107	1.057349	0.814458	1.1634	1.270368	1.246399	0.98059	0.7168	0.641917	0.532715	0.465466
OA003	0.035302	0.035691	0.047971	0.051797	0.048435	0.071822	0.116564	0.535782	1.200677	1.490537	0.978002	0.719873	0.485756	0.526644	0.424038	0.335529	0.313568	0.328381	0.427293	0.395037
OA004	0.020355	0.022919	0.026665	0.028449	0.030776	0.043133	0.112605	0.348199	0.469154	0.541484	0.542347	0.47829	0.341301	0.219018	0.15692	0.153958	0.176368	0.231154	0.222621	0.186649
OA005	0.025214	0.031402	0.051892	0.059052	0.079752	0.1029	0.240677	0.708774	1.054611	1.500788	1.144142	0.897959	0.66473	0.567255	0.474144	0.394324	0.414346	0.469327	0.475849	0.462525
OA006	0.011868	0.017274	0.020049	0.019755	0.01723	0.022503	0.066901	0.192159	0.561654	0.64087	0.580941	0.379679	0.298271	0.326845	0.316824	0.277322	0.25957	0.246929	0.230147	0.17698
OA007	0.019527	0.016124	0.015984	0.017142	0.019826	0.057962	0.224007	0.48784	0.686202	0.619274	0.554308	0.47571	0.514345	0.53591	0.445439	0.514666	0.506961	0.400853	0.391867	0.406304
OA008	0.022291	0.026907	0.030486	0.029795	0.038761	0.081009	0.156927	0.337015	0.695266	0.949261	1.030793	0.83484	0.77193	0.648915	0.386287	0.346374	0.28527	0.206427	0.175062	0.117263
OA009	0.007912	0.009751	0.007756	0.009484	0.013979	0.016957	0.039785	0.74308	0.607352	0.485911	0.443928	0.404733	0.564286	0.631679	0.427717	0.24771	0.263982	0.282398	0.293239	0.308372
OA010	0.028501	0.025869	0.044713	0.04884	0.076056	0.089956	0.225418	0.398385	0.365388	0.474429	0.495963	0.611597	0.617501	0.524847	0.539748	0.54437	0.492655	0.393672	0.384512	0.397078
OA011	0.036664	0.036085	0.061568	0.076575	0.105265	0.155526	0.254911	0.438478	0.683545	0.597103	0.693553	0.598051	0.560131	0.430118	0.501266	0.389747	0.347037	0.290578	0.235144	0.265115
OA012	0.059749	0.092258	0.117625	0.104107	0.113056	0.228407	0.525688	0.813365	1.100619	0.942721	0.935441	0.791431	0.770446	0.886087	0.923232	0.841675	0.866953	0.54749	0.603459	0.600326
OA013	0.012642	0.015409	0.015703	0.015215	0.018009	0.065035	0.549278	0.924077	0.758957	0.735111	0.763114	0.608104	0.707748	0.873379	0.783362	0.760718	0.498642	0.358927	0.247748	0.169847
Mean	0.027617	0.032437	0.041255	0.043702	0.054614	0.090561	0.256597	0.56692	0.756238	0.823345	0.759489	0.63588	0.626352	0.611604	0.544785	0.464953	0.406964	0.346949	0.334056	0.312145
Standard deviation	0.01786	0.024856	0.031169	0.029783	0.035164	0.060856	0.189635	0.274051	0.29052	0.362819	0.231175	0.162673	0.20961	0.260265	0.273542	0.243011	0.200524	0.139599	0.141049	0.149025

Control group	0～5%	5～10%	10～15%	15～20%	20～25%	25～30%	30～35%	35～40%	40～45%	45～50%	50～55%	55～60%	60～65%	65～70%	70～75%	75～80%	80～85%	85～90%	90～95%	95～100%
Cont001	0.017381	0.021843	0.023109	0.029606	0.024746	0.052286	0.240478	0.540337	0.553576	0.619308	0.586852	0.379041	0.328676	0.328488	0.354453	0.39779	0.40927	0.456319	0.422682	0.413099
Cont002	0.010564	0.013275	0.013205	0.015667	0.018823	0.023599	0.049388	0.247494	0.760394	0.692241	0.567335	0.477555	0.52621	0.573567	0.621657	0.573188	0.616654	0.602534	0.568291	0.518047
Cont003	0.075174	0.06133	0.070915	0.07637	0.089341	0.104457	0.154952	0.212221	0.304958	0.264738	0.300442	0.179708	0.180517	0.190455	0.139041	0.128978	0.101346	0.095774	0.100308	0.095533
Cont004	0.017766	0.025231	0.030603	0.040603	0.046154	0.049589	0.228739	0.620995	0.964963	0.951063	0.860277	0.601617	0.494502	0.470648	0.488699	0.419769	0.354413	0.30718	0.298171	0.223841
Cont005	0.010922	0.01669	0.02209	0.030139	0.030306	0.039787	0.057343	0.253616	0.5366	0.546636	0.544918	0.462144	0.374848	0.356776	0.354951	0.361056	0.310186	0.22927	0.18999	0.153859
Cont006	0.021242	0.029366	0.03733	0.038879	0.063494	0.060579	0.078467	0.47133	0.816432	0.929752	0.716037	0.62834	0.503429	0.364317	0.313396	0.250818	0.220663	0.234322	0.190696	0.167774
Cont007	0.063763	0.063502	0.050202	0.068928	0.080045	0.08644	0.11336	0.117633	0.212485	0.431027	0.153734	0.097552	0.118875	0.178762	0.226497	0.258091	0.273974	0.30763	0.340618	0.37078
Cont008	0.051906	0.075697	0.08589	0.075617	0.098405	0.095197	0.236944	0.54097	1.591641	1.807532	1.865396	1.601877	1.193779	0.940345	1.02371	0.913994	0.933489	0.961639	0.66567	0.430311
Cont009	0.020483	0.022503	0.027849	0.03815	0.041775	0.043553	0.051802	0.204591	0.608398	0.936745	0.991985	0.874734	0.616924	0.532898	0.409983	0.36615	0.288008	0.251078	0.192629	0.1392
Cont010	0.010035	0.012951	0.01328	0.016558	0.013991	0.017271	0.045743	0.295985	0.56285	0.602659	0.540883	0.390959	0.297562	0.256546	0.204318	0.172905	0.086158	0.066128	0.055857	0.061243
Cont011	0.023589	0.04147	0.05128	0.043971	0.048873	0.058673	0.148939	0.44575	0.775922	0.70724	0.69371	0.495577	0.390719	0.376548	0.305626	0.292341	0.286307	0.242898	0.203468	0.154573
Mean	0.029348	0.034896	0.038705	0.043135	0.050541	0.057403	0.127832	0.359175	0.698929	0.771722	0.711052	0.562646	0.456913	0.415395	0.403848	0.375917	0.35277	0.341343	0.293489	0.248024
Standard deviation	0.021978	0.021221	0.022473	0.020644	0.027552	0.026697	0.075224	0.161227	0.351265	0.386515	0.427027	0.385087	0.273125	0.205467	0.234124	0.206677	0.229316	0.242414	0.182486	0.148651

d) Rectus femoris

Knee OA Group	0〜5%	5〜10%	10〜15%	15〜20%	20〜25%	25〜30%	30〜35%	35〜40%	40〜45%	45〜50%	50〜55%	55〜60%	60〜65%	65〜70%	70〜75%	75〜80%	80〜85%	85〜90%	90〜95%	95〜100%
OA001	0.025538	0.028677	0.031151	0.039354	0.035034	0.038782	0.058413	0.09588	0.164296	0.240389	0.256285	0.219729	0.230496	0.184062	0.151229	0.138131	0.108415	0.110666	0.098337	0.093739
OA002	0.041808	0.053951	0.096365	0.190555	0.155957	0.22285	0.309812	0.471226	0.64852	0.710947	0.540871	0.198838	0.176961	0.183333	0.171291	0.149246	0.136807	0.114387	0.098811	0.092041
OA003	0.038007	0.04215	0.059626	0.058271	0.062422	0.078889	0.114826	0.2909	0.562437	0.829858	0.5584	0.360179	0.259062	0.272515	0.304664	0.333578	0.335789	0.360118	0.413881	0.396388
OA004	0.032655	0.038448	0.044185	0.054753	0.04674	0.044938	0.04654	0.079643	0.087005	0.075245	0.086302	0.053866	0.053953	0.048345	0.054167	0.076068	0.099916	0.109063	0.127232	0.117405
OA005	0.018229	0.020103	0.022273	0.025398	0.027525	0.031769	0.078408	0.188039	0.460643	0.511372	0.289859	0.185618	0.152484	0.129508	0.132434	0.128502	0.158195	0.165166	0.154076	0.158909
OA006	0.012247	0.015483	0.014342	0.014678	0.013359	0.038457	0.066165	0.086798	0.305342	0.372579	0.229651	0.095689	0.101951	0.113916	0.114177	0.102111	0.113975	0.103795	0.114087	0.082936
OA007	0.05104	0.082259	0.09121	0.115187	0.109423	0.144752	0.156357	0.287131	0.398713	0.316127	0.239259	0.16874	0.180764	0.167963	0.153615	0.199006	0.230353	0.183296	0.193039	0.179181
OA008	0.067631	0.072705	0.087855	0.111638	0.119433	0.126133	0.126527	0.177302	0.34826	0.479704	0.502437	0.342439	0.263808	0.196454	0.197004	0.209101	0.208386	0.183846	0.200318	0.160518
OA009	0.014055	0.029475	0.03097	0.042303	0.038089	0.042158	0.06623	0.232871	0.261139	0.263875	0.249457	0.148865	0.150199	0.173048	0.188378	0.176637	0.183853	0.176394	0.191908	0.18458
OA010	0.048371	0.051227	0.069163	0.069982	0.086193	0.139247	0.459291	0.881518	0.799414	0.988943	0.853571	0.59221	0.458429	0.390108	0.340794	0.36657	0.381173	0.362995	0.385223	0.342087
OA011	0.034189	0.043742	0.047655	0.052955	0.058302	0.103007	0.162077	0.236295	0.28847	0.284493	0.239659	0.181568	0.12714	0.119023	0.121633	0.123519	0.12024	0.118579	0.10918	0.114126
OA012	0.031669	0.04217	0.063511	0.06263	0.051797	0.05676	0.102669	0.217628	0.326155	0.410864	0.349441	0.182782	0.174638	0.185695	0.176208	0.186536	0.20518	0.143715	0.142064	0.130451
OA013	0.066195	0.070778	0.085348	0.095571	0.136267	0.171866	0.340955	0.393239	0.621103	0.714703	0.675686	0.465354	0.263166	0.224803	0.199821	0.207833	0.183355	0.188696	0.152784	0.127447
Mean	0.037049	0.045475	0.057204	0.07179	0.072349	0.095354	0.160636	0.279883	0.4055	0.476854	0.390068	0.245837	0.199465	0.183752	0.17734	0.184372	0.189664	0.178517	0.183149	0.167678
Standard deviation	0.01714	0.019603	0.02674	0.045057	0.043224	0.058897	0.123622	0.205647	0.196544	0.254203	0.209918	0.146743	0.096793	0.079914	0.073121	0.081142	0.082898	0.083793	0.09832	0.092004

Control group	0〜5%	5〜10%	10〜15%	15〜20%	20〜25%	25〜30%	30〜35%	35〜40%	40〜45%	45〜50%	50〜55%	55〜60%	60〜65%	65〜70%	70〜75%	75〜80%	80〜85%	85〜90%	90〜95%	95〜100%
Cont001	0.034745	0.039669	0.043214	0.04731	0.042804	0.037602	0.115779	0.211347	0.279411	0.34912	0.40335	0.16796	0.092303	0.0907	0.089026	0.11427	0.127735	0.121284	0.129318	0.137784
Cont002	0.028209	0.030599	0.036425	0.044233	0.051594	0.058875	0.039306	0.059581	0.231541	0.379814	0.439964	0.327291	0.252615	0.242964	0.197507	0.230291	0.248242	0.266599	0.246651	0.244831
Cont003	0.053217	0.055427	0.073835	0.087235	0.09605	0.125507	0.333869	1.019244	1.680043	1.503718	1.579891	1.016352	0.702504	0.476997	0.353479	0.226773	0.147945	0.140103	0.129778	0.123255
Cont004	0.042476	0.05206	0.075246	0.102784	0.120278	0.112749	0.222031	0.292671	0.371878	0.253285	0.27163	0.115747	0.088878	0.09526	0.108748	0.106337	0.105974	0.094804	0.099445	0.099706
Cont005	0.031545	0.04552	0.058182	0.103841	0.139945	0.208639	0.240694	0.24328	0.32938	0.357067	0.292339	0.181078	0.104202	0.0771	0.06987	0.075866	0.070828	0.071966	0.069396	0.06287
Cont006	0.018232	0.017998	0.021631	0.021738	0.036993	0.031273	0.030339	0.083309	0.168978	0.254561	0.170981	0.120894	0.103479	0.091928	0.082986	0.073348	0.085448	0.091526	0.072843	0.069703
Cont007	0.031833	0.038299	0.044866	0.054406	0.054128	0.052547	0.142721	0.405676	0.674038	0.794827	0.694273	0.59295	0.644145	0.851095	0.827189	0.803517	0.776667	0.888179	0.895809	0.944978
Cont008	0.016569	0.020248	0.019241	0.019462	0.02197	0.033019	0.055923	0.112173	0.272011	0.32969	0.287781	0.201315	0.14142	0.130733	0.150421	0.154072	0.167457	0.187197	0.132031	0.103233
Cont009	0.013323	0.014758	0.018494	0.021758	0.024881	0.021984	0.029736	0.058612	0.150582	0.182196	0.212637	0.18434	0.168814	0.152001	0.160574	0.138699	0.137711	0.141419	0.116844	0.112533
Cont010	0.015054	0.017528	0.017932	0.02063	0.01894	0.016795	0.019188	0.123565	0.269646	0.27811	0.214087	0.112564	0.111402	0.122251	0.119681	0.11464	0.071345	0.062983	0.055127	0.067119
Cont011	0.021388	0.0266	0.029845	0.03134	0.032037	0.041714	0.060486	0.15116	0.268538	0.322227	0.245569	0.140283	0.091876	0.117596	0.124238	0.135515	0.136299	0.142223	0.125023	0.125621
Mean	0.027872	0.03261	0.039901	0.050431	0.058147	0.067337	0.117279	0.250965	0.426913	0.454965	0.4375	0.287343	0.227421	0.222602	0.207611	0.197575	0.188696	0.200753	0.188388	0.190149
Standard deviation	0.01195	0.013818	0.020417	0.031434	0.039731	0.055868	0.10076	0.263671	0.417849	0.364524	0.387475	0.266172	0.215461	0.226939	0.20971	0.197872	0.192022	0.224162	0.228881	0.243411
