# Supplementary material for: Dynamic Frequency Analyses of Lower Extremity Muscles during Sit-To-Stand Motion for the Patients with Knee Osteoarthritis
Source: PLoS One. 2016 Jan 25;11(1):e0147496. doi: 10.1371/journal.pone.0147496 (PMC4726819; doi:10.1371/journal.pone.0147496)
Supplement: S4 Table — (PDF) [file pone.0147496.s004.pdf]

S4 Table. The detailed data of mean power frequency changes of each muscle of the knee OA group and the control group.

a) Gluteus maximus

Knee OA Group	35–40%	40–45%	45–50%	50–55%	55–60%	60–65%	65–70%	70–75%	75–80%	80–85%	85–90%	90–95%	95–100%
OA001	30	40.66667	52.6	90.6	84	68.2	102.8	103.8	111.2	115.2	122.6	116.6	112
OA002		47.5	49.5	57.8	96.4	116.8	133.8	145.8	159.6	142.6	141.8	133.6	150.2
OA003	35.5	48.6	51.2	76.2	95.4	92.6	114.8	88.4	93.2	106.4	127.8	109	105.6
OA004	32.75	36.4	53.4	63.2	74.6	76.2	82.6	91.2	86.5	81.25	63	39	53
OA005	61.33333	85	65	113.6	113.4	111.6	117.8	121.6	117.4	117.6	129.2	117	138.8
OA006		40.5	40.6	77.4	90.2	144.4	129	131.8	135.6	100	170.6667		
OA007		30	81	39	84.33333	95	105.5	89.5	110	101.6667	137	35	
OA008					110.3333	128.75	80	134.2	149.4	142.8	139.2	116.4	112.8
OA009	58.2	56.2	59.6	89.2	113	92.4	114	109.6	136.2	109.25	120.5		125
OA010	108.6667	109.5	104.75	100.2	116.6	139.2	140.8	133.4	144.4	161.4	174.8	162.4	143.2
OA011	39	46.6	46.4	80.8	71.4	64	73.4	94.8	92.2	109	119.2	121	107.8
OA012	57.33333	48.6	54	76.8	92	111	120	125.6	117.2	100.2	109.2	110.4	112
OA013			52	46.5	45.8	47.2	72.2	90	105.2	121.2	118.6	106.4	100.8
Mean	52.84792	53.59697	59.17083	75.94167	91.34359	99.02692	106.6692	112.2846	119.8538	116.0436	128.7359	106.0727	114.6545
Standard deviation	24.12252	22.24946	16.85181	20.70984	19.32143	28.63442	22.22782	19.82624	22.4599	20.79672	26.64821	35.76146	25.06009

Control group	35–40%	40–45%	45–50%	50–55%	55–60%	60–65%	65–70%	70–75%	75–80%	80–85%	85–90%	90–95%	95–100%
Cont001	77.4	97	77.2	123.4	151	157.2	145.4	131	138.8	162.2	135.8	161.6	167.4
Cont002	40	90.4	60.6	94.2	100.8	111.2	86	105	92.8	115.4	93.2	111.8	103.2
Cont003	71.5	75.8	59.25	80.6	104.4	113.8	105.4	117.2	112.2	124.75	100.5	129.25	126
Cont004	33.5	43.25	48.75	90.6	86.8	78.8	104	106	109.4	131	125.75	120.25	111
Cont005	23.4	58	33.6	86.2	119.2	123.8	128.8	117.2	108	111.6	130.8	115.6	130.2
Cont006	37.8	62.8	52.6	71.8	88	92.4	78.4	72	88.4	85.6	86	101	94
Cont007	38	40.8	47.4	66.8	83.6	82	86.2	112.4	116	138.4	158.2	85.5	156.6667
Cont008	28	62.5	57	89.4	119.4	132.4	157.4	134.6	139.6	134.8	146.8	148.6	168.8
Cont009		62	49.2	75	76.4	100.6	91.4	87.2	126.2				
Cont010	29.2	38	26.4	70.2	77.2	77.2	81.8	92.2	78	114.2	76.5		
Cont011	38	34.4	43.8	84.6	96.25	97.6	86.4	101.2	117.6	88.4	121.5	116	64
Mean	41.68	60.45	50.52727	84.8	100.2773	106.0909	104.6545	106.9091	111.5455	120.635	117.505	121.0667	124.5852
Standard deviation	17.18743	19.88884	13.01673	14.93915	21.41201	23.65085	25.99889	17.70883	18.66238	21.87521	25.81388	21.80152	33.49744

b) Medial hamstrings

Knee OA Group	35–40%	40–45%	45–50%	50–55%	55–60%	60–65%	65–70%	70–75%	75–80%	80–85%	85–90%	90–95%	95–100%
OA001	64.5	141	82	82.6	127.6	143.2	157.2	171.8	193	199.6	188.4	175	211
OA002	74.2	70.2	57.4	147.6	169.8	165.4	173	172.8	190	196.4	206.8	188.6	181.4
OA003	44	65.8	111.6	123.6	126.8	167	177.8	177.8	146.8	174.8	183.2	162.6	183.2
OA004	62	52	63.8	76.4	74.6	97.4	104.8	113.6	121.6	131.2	136	148	140
OA005	68.4	81.2	89.4	115.4	162.6	157.8	161	192.8	177.8	193.6	192.4	193.6	186.2
OA006	64.25	66.8	56.6	97.2	159	185.2	200.8	200.4	181	201.2	200.4	184.2	168.8
OA007	43.33333	44	52	78	149	112.8	136.8	156.4	136.4	146	156	89	
OA008	106.5	98.8	112	134.2	130	131.6	146.6	149.4	164	170	190.6	188.8	181.8
OA009	56.4	59.6	68.4	104.2	131.4	162	165	181.8	193	192.2	148.6	195	179
OA010	153.2	157.2	142	146.2	160.8	175.8	171.8	187.4	203.4	215	222.6	184.6	220
OA011	48.33333	48.25	50	97	99.8	122.2	123.8	153	154.8	174	163.2	157.8	178.8
OA012	61.2	77.8	50.2	95	101.6	117.6	139.6	156.8	168	157.6	175	121.2	173
OA013	26.5	44.66667	54.2	77.4	51.6	56.8	86.2	135.8	144.2	153.8	164.2	173.8	184
Mean	67.13974	77.4859	76.12308	105.7538	126.5077	138.0615	149.5692	165.3692	167.2308	177.3385	179.0308	166.3231	182.2667
Standard deviation	30.76818	34.17486	28.36103	24.56942	34.63121	34.89222	30.18791	23.37373	24.18224	23.9518	23.75954	30.11501	19.03091

Control group	35–40%	40–45%	45–50%	50–55%	55–60%	60–65%	65–70%	70–75%	75–80%	80–85%	85–90%	90–95%	95–100%
Cont001	38.75	39.4	53.2	73.4	114.6	108.8	134.6	141.6	156	158.6	165.4	146.4	156.4
Cont002	91.6	60.2	65.6	84.8	110.8	143	124.2	114.4	117.6	137.4	140.6	161.2	166.5
Cont003	32.5	48	52.75	86.8	102.6	126.4	170.2	174.2	137	160	158.5	165.5	178
Cont004	39.6	48	53.2	119.2	120.8	140.2	152.2	133.4	132	152.5	149.3333	151	219
Cont005	45	67.8	82.6	111.2	114.2	138.6	151.8	159.2	195.4	163.6	212	177.6	179.6
Cont006	67.4	53.8	73.8	70.2	101.8	126.6	135.8	135.4	111.2	138.8	141	138.2	157.25
Cont007	44	57	70	103.8	110	120	133.2	138.4	157.2	168.4	159.8	165.6	152
Cont008	105	69.6	110.6	156.4	144.4	149.6	155.8	181.6	166	178.8	190	162.6	189.5
Cont009		73	77.4	107.8	135.8	148	172.6	185	174.4	158.4	172.6	174.4	175.4
Cont010	64.5	59.6	62.4	110.6	153.2	154.2	160.2	151.8	168	157.4	165.8	155.4	128.75
Cont011	60.4	52.6	59.6	89.2	114.2	126.6	122.8	149.2	148.4	149	132.4	162.75	158.75
Mean	58.875	57.18182	69.19545	101.2182	120.2182	134.7273	146.6727	151.2909	151.2	156.6273	162.4939	160.0591	169.1955
Standard deviation	22.78387	9.767275	16.28307	23.28456	16.13138	13.41811	16.70411	20.97745	24.04405	11.52295	22.09963	11.10616	22.30236

c) Vastus medialis

Knee OA Group	35−40%	40−45%	45−50%	50−55%	55−60%	60−65%	65−70%	70−75%	75−80%	80−85%	85−90%	90−95%	95−100%
OA001	73.8	79.8	89.8	106.6	113	117.6	113	125.4	141	150.2	140.8	143.4	178
OA002	64.4	74.4	73.6	143.2	159.8	156.4	161.2	173	169	196.6	165.2	163.2	159
OA003	51	57.8	82.6	91.8	105.4	125.2	121.8	128.8	129.4	145.2	135	151.6	159
OA004	89.4	84.6	74.4	93.6	95.6	116	111.4	140	133.6	132.4	150.2	138.8	131.6
OA005	62.6	79.8	85.2	122.6	137	156.8	165.8	144.6	169.4	165.8	174	166.4	187.8
OA006	86.2	77.4	72.6	112.6	166.8	184.8	153	179.4	181.4	199.2	179	199.4	174
OA007	62.8	82.4	67.2	104	112.2	136	120.6	137	141.6	138	148.8	130.2	149.4
OA008	87.6	102.8	98.4	117	143.6	144.2	143.2	164.2	156.8	176	174.2	173.2	153.6
OA009	26.8	63	60.2	108.6	133.2	131	138.8	163.2	181.4	151.4	160.2	161.2	160
OA010	143	140.8	181.4	170.6	158	174.4	161	188	189	179.2	229.4	197.2	203.6
OA011	74.2	71.8	60.4	106	111.4	106	105.2	129.2	120.8	135	139.6	134.2	154
OA012	74.2	84.4	77.8	114.6	131.4	128.4	125.6	129.8	126.6	125	138	134.8	118.8
OA013		40.33333	55.2	55.8	60.8	79.4	110.4	132.4	142.4	137.4	130.6	150.6	138.4
Mean	74.66667	79.94872	82.98462	111.3077	125.2462	135.0923	133.1538	148.8462	152.4923	156.2615	158.8462	157.2462	159.0154
Standard deviation	26.50652	22.74178	30.78656	25.70754	28.43023	27.46025	20.8964	20.93788	22.35829	23.77515	25.66712	21.72757	22.08911

Control group	35−40%	40−45%	45−50%	50−55%	55−60%	60−65%	65−70%	70−75%	75−80%	80−85%	85−90%	90−95%	95−100%
Cont001	61.6	67.2	68.8	107.4	127.4	123.6	125.2	136.6	141.6	140.4	142	154.4	143.6
Cont002	50	71	65.2	99.8	106.4	116.6	110.2	119	123.6	142	156	137	129.6
Cont003	83.2	103.4	81.6	128	137.8	122.4	154.6	141.6	134.6	124.4	167	120.2	154.8
Cont004	54	80.8	82.2	121.2	151.4	134.4	135.4	150.2	147.8	136.8	143.4	137.6	143.6
Cont005	46.4	66.4	64	113.6	154.6	147.4	143.8	142	156.8	156	159.8	146.6	171.8
Cont006	25.8	68.2	82.8	104.2	112.6	129.6	130.4	122.8	119.6	130.8	136	157.8	124.4
Cont007	73.4	77.6	73	88.8	110.2	112.4	128.6	151.8	164.2	155	147.4	148.6	145.4
Cont008	74.5	85.8	91.4	132.4	139	158.8	160.8	164.4	148	167.2	176.4	179.8	169.8
Cont009	41	85.8	105.6	119	132.4	146.4	149.2	138.6	161.6	160	176.4	174	174
Cont010	57.4	63	76.4	94.2	112.4	106	127.2	133.6	143.6	129.4	136.2	128.2	137.6
Cont011	46.75	68	64	92.4	113	101.4	104	113.6	122.6	129.6	136.6	114.6	147.8
Mean	55.82273	76.10909	77.72727	109.1818	127.0182	127.1818	133.5818	137.6545	142.1818	142.8727	152.4727	145.3455	149.3091
Standard deviation	15.87619	11.5079	12.29258	14.11342	16.43222	17.36066	16.79274	14.37475	14.89196	13.79388	14.85696	19.67612	15.93814

d) Rectus femoris

Knee OA Group	35–40%	40–45%	45–50%	50–55%	55–60%	60–65%	65–70%	70–75%	75–80%	80–85%	85–90%	90–95%	95–100%
OA001	119	95.2	95	153.6	128.6	160.8	138.6	144	154.2	171.2	185.4	174.4	190.4
OA002	97	107	92.2	160	175	169.6	167.8	163.4	194.2	181.8	197.4	212.8	169.4
OA003	57	59.6	72.8	108.6	104.6	150.2	132.4	144.2	150.2	153.6	161	177.8	154.8
OA004	46.4	97.8	86.4	102.4	100.8	119.6	123.2	135	158.6	142.4	149.6	160.8	161.2
OA005	67.2	89.2	87	143	168.4	158.2	167	163.4	174	175.8	184.2	199.8	201.6
OA006	83	90.4	91.4	135.6	170.8	164.4	185.2	197.6	186.8	203.8	184	196	191.2
OA007	107	98.8	100.8	104.4	133.8	152.2	149	157	172.2	143.8	179	165.8	171
OA008	88.4	130.4	132.8	167.8	181.4	152.4	147.2	172.6	177.4	198.6	191.4	180.6	181.2
OA009	75.6	86.6	75.6	124.6	143.6	140.2	151	150	171	182.2	189	178.4	161.4
OA010	184	191.6	214.2	194.6	221	199.8	192	209	199.2	195.4	246.2	219.8	252.4
OA011	126	85.6	97.2	129.2	132.2	131.8	111.4	131.4	133.8	138	170.8	157.8	144.8
OA012	74.8	87.8	127	144.4	142.2	152.6	126.2	164	161.8	149.2	156.8	167.4	153.4
OA013	91.4	92.6	74	97.6	90	83.8	131	128.8	156.4	141.8	149.6	160.8	168.4
Mean	93.6	100.9692	103.5692	135.8308	145.5692	148.8923	147.8462	158.4923	168.4462	167.5077	180.3385	180.9385	177.0154
Standard deviation	34.13124	30.14364	36.38925	27.70636	35.506	26.29824	23.42545	23.22818	17.7591	22.89946	24.48024	19.46617	26.94227

Control group	35–40%	40–45%	45–50%	50–55%	55–60%	60–65%	65–70%	70–75%	75–80%	80–85%	85–90%	90–95%	95–100%
Cont001	83	96.8	80.8	116.8	124.2	125.8	143.4	161	155	163.2	166	182.8	175.8
Cont002	130	66.2	113.8	145.8	151.8	168	153.2	126.8	148.8	161.6	157	148	142
Cont003	97	85.4	80.6	115	138.6	129.6	152.8	143.2	138.4	148.8	110.25	147	180
Cont004	80.4	88	111.8	136.8	163.4	141.8	127	130.2	147.2	153.4	162.4	147.4	156.2
Cont005	92.2	82.4	90.6	143.6	185	176	146.6	166.4	163.6	188.6	174.4	168.2	194.8
Cont006	42.25	55.2	81.4	141.4	121.6	96	127.2	129.4	120.4	144.4	141.8	137.6	159.8
Cont007	35.8	58.2	50.4	98	97.8	111	114.2	116.2	127	116.8	122.6	123.2	125.2
Cont008	84.5	76.4	102	139.8	165.8	160	160.6	186.6	174	186.8	200.2	181.4	191.8
Cont009	76.5	85.4	98.4	113.6	136.8	154.2	176.8	174.2	172.6	171.2	163.6	202.4	172.2
Cont010	45	62.6	88	128.4	124.4	132.2	130.6	119.2	153.6	143	149.4	145.8	143
Cont011	51.6	66.6	78.2	127.8	111.2	107.8	100.8	144.2	147.4	148.8	142.6	158.4	151.4
Mean	74.38636	74.83636	88.72727	127.9091	138.2364	136.5818	139.3818	145.2182	149.8182	156.9636	153.6591	158.3818	162.9273
Standard deviation	27.04058	13.11843	17.04844	14.64225	24.85826	24.75486	20.86989	22.54135	16.13882	19.69325	23.48118	22.04532	20.97397
