# Supplementary material for: Dynamic Frequency Analyses of Lower Extremity Muscles during Sit-To-Stand Motion for the Patients with Knee Osteoarthritis
Source: PLoS One. 2016 Jan 25;11(1):e0147496. doi: 10.1371/journal.pone.0147496 (PMC4726819; doi:10.1371/journal.pone.0147496)
Supplement: S5 Table — (PDF) [file pone.0147496.s005.pdf]

S5 Table. The detailed data of the rate of each frequency band for rectus femoris of the knee OA group and the control group.

Lower frequency band (0-45 Hz)

Knee OA Group	35-40%	40-45%	45-50%	50-55%	55-60%	60-65%	65-70%	70-75%	75-80%	80-85%	85-90%	90-95%	95-100%
OA001	55.48173	62.02532	45.9605	44.35424	43.65163	24.83157	29.6875	26.88312	28.54478	18.7643	23.19277	22.92994	20.56962
OA002	51.37649	50.56581	49.36664	36.38738	28.80686	17.64113	26.1633	16.94915	16.03631	18.54839	18.75	22	16.99779
OA003	76.97044	67.18995	66.84701	58.10405	34.85017	27.63038	24.34095	25.50274	20.48544	17.80944	15.70482	14.56954	20.70447
OA004	66.84492	56.27148	58.08241	54.90549	48.57143	44.77612	49.55595	33.4608	32.1608	20.75665	30.53097	24.93333	27.77778
OA005	62.04188	58.3228	55.42694	35.21227	28.44444	21.34831	19.55923	12.6498	22.79412	24.32859	8.844765	14.68144	19.6129
OA006	44.55782	48.11189	53.81744	44.43905	29.6729	20.33426	21.35338	17.99308	13.96104	14.1604	15.85821	12.44076	23.67816
OA007	53.25	58.31933	54.80902	52.19917	41.15334	35.95342	29.86799	30.19197	27.38739	31.91489	25.16447	25.45757	27.17718
OA008	50.23772	51.71806	48.09671	32.04837	22.72727	29.19847	28.51124	19.23077	24.13194	21.02351	15.59934	24.05797	15.66901
OA009	66.71501	58.64023	59.44112	40.48938	30.5303	29.89922	35.90535	28.32653	28.64823	23.16684	24.90909	20.21196	20.92624
OA010	37.59011	30.3217	23.31014	20.4936	18.67347	18.75	14.46886	20.96289	12.48581	14.93827	9.626719	7.588076	13.37047
OA011	45.89759	58.53895	50.31139	36.77494	34.14634	33.50297	43.75716	41.17647	28.87168	26.47815	32.55474	33.43849	26.67785
OA012	65.00542	59.64153	46.36634	35.55397	45.9069	32.19731	27.08499	32.21406	22.2097	22.34332	24.1012	23.05476	38.28756
OA013	62.841704	62.392044	66.244783	42.782987	29.123196	37.95972	37.12297	35.075988	23.557188	33.992095	25.44757	27.586207	25.138889
Mean	56.8316	55.54301	52.16003	41.0573	33.55833	28.77099	29.79837	26.20134	23.17496	22.17114	20.79113	20.99616	22.81446
Standard deviation	10.6045	8.799702	10.57814	9.769404	8.642422	7.820878	9.361705	7.970686	5.87839	5.700615	7.105916	6.71834	6.211317

Control group	35-40%	40-45%	45-50%	50-55%	55-60%	60-65%	65-70%	70-75%	75-80%	80-85%	85-90%	90-95%	95-100%
Cont001	68.9747	55.64388	63.82506	48.21147	41.28837	41.16223	29.27273	35.12786	21.82891	20.43011	26.01399	19.9095	18.47969
Cont002	69.30091	71.25551	55.43033	37.67158	36.40705	30.10949	24.45194	44.20328	35.38826	26.20434	33.87246	30.63255	34.04727
Cont003	66.63573	53.42131	63.22017	46.60377	35.17754	32.99857	29.061	28.27521	32.25806	14.0665	22.41993	20.84806	22.64151
Cont004	52.52905	65.62794	57.35939	39.80986	34.00317	38.12677	28.41823	28.40495	26.46388	31.05745	20.94361	22.76657	27.81513
Cont005	67.99403	55.76094	56.97273	39.81406	21.875	28.08431	23.56995	24.69586	21.05978	21.92882	21.07905	25.20868	21.68525
Cont006	81.77172	75.70423	73.24823	52.33031	43.26439	50.4127	35.62753	33.50831	36.20072	31.00671	39.66725	33.40564	27.38496
Cont007	80.02183	78.66692	80.06255	52.86238	49.52662	41.31503	34.21953	42.08561	28.6444	38.87308	33.15059	24.42895	32.07769
Cont008	54.19847	60.3352	51.19435	41.0609	24.13059	23.45815	31.44105	19.58042	18.22985	11.66181	15.65585	14.65753	13.17992
Cont009	51.19048	54.0412	53.40573	36.79144	26.03116	28.66756	22.31076	24.34402	22.55747	18.83803	18.30743	13.66337	20.68311
Cont010	72.07207	60.47672	68.14048	47.86074	34.65347	29.24901	29.39371	34.73242	23.33614	25.68543	27.88671	23.96694	19.00958
Cont011	74.05858	71.38024	59.60265	40.81187	39.19598	45.20325	37.81388	26.53928	27.04327	23.10606	35.6091	25.62574	24.71627
Mean	67.15887	63.84673	62.04197	43.9844	35.0503	35.34428	29.59821	31.0452	26.63734	23.89621	26.78236	23.19214	23.79276
Standard deviation	9.984473	8.719086	8.429516	5.504118	8.036393	8.034701	4.732502	7.279888	5.691395	7.526172	7.494476	5.655912	5.899984

Middle frequency band (46–80 Hz)

Knee OA Group	35–40%	40–45%	45–50%	50–55%	55–60%	60–65%	65–70%	70–75%	75–80%	80–85%	85–90%	90–95%	95–100%
OA001	19.93355	17.24684	23.42908	16.90037	19.20252	21.17421	17.52232	22.85714	11.3806	13.50114	20.18072	19.10828	22.46835
OA002	22.47024	21.62001	22.47993	18.86834	16.54581	18.85081	13.34504	22.35169	11.95159	11.45161	12.32639	12.66667	15.23179
OA003	13.05419	19.15228	16.44124	18.0578	31.8535	23.33028	26.44991	23.67459	22.03883	23.50846	20.5298	19.42605	22.16495
OA004	19.65241	16.66667	17.67036	22.68227	24.41558	23.43284	15.98579	22.56214	14.82412	26.17587	13.79056	14.66667	23.28767
OA005	22.25131	21.4075	19.63947	15.73314	18.22222	19.20327	24.93113	25.2996	24.44853	13.11216	13.80866	14.40443	15.6129
OA006	29.2517	25.17483	18.66305	15.63866	17.64019	21.44847	13.08271	15.74394	12.82468	16.79198	15.85821	13.50711	14.71264
OA007	22.25	18.48739	19.46618	24.06639	18.0865	27.2198	19.14191	24.78185	25.04505	21.74941	14.96711	18.96839	16.21622
OA008	20.12678	21.32159	17.59259	20.93726	24.76255	18.22519	12.7809	24.92308	17.01389	14.93776	19.37603	11.01449	17.60563
OA009	16.4612	19.4051	21.47705	23.08403	18.78788	24.86002	18.107	20.16327	13.28725	21.67906	21.36364	15.21575	19.21098
OA010	18.33162	17.21424	14.76143	19.99375	8.316327	19.11765	10.16484	13.44032	7.491487	9.382716	7.956778	9.123758	11.42061
OA011	23.5657	22.68989	22.15302	18.7355	27.58621	26.20865	23.25315	21.56863	28.09735	27.50643	21.31387	12.46057	21.14094
OA012	15.05959	16.68727	21.63196	25.68579	18.61958	23.13901	20.96902	17.20881	19.16573	21.07175	21.57124	16.13833	15.34733
OA013	21.805467	16.644857	15.524438	18.45278	28.923418	18.432574	24.303944	17.81155	21.301154	16.442688	16.368286	16.61442	19.166667
Mean	20.32414	19.5168	19.30229	19.91047	20.9971	21.8956	18.46444	20.95282	17.6054	18.2547	16.87779	14.87038	17.96821
Standard deviation	3.966542	2.622013	2.69792	3.066249	6.024176	2.936848	5.060144	3.651743	6.016633	5.535046	4.081081	3.050201	3.461688

Control group	35–40%	40–45%	45–50%	50–55%	55–60%	60–65%	65–70%	70–75%	75–80%	80–85%	85–90%	90–95%	95–100%
Cont001	16.24501	23.6354	18.71757	17.41189	25.55123	23.36562	28.18182	18.43876	23.30383	18.63799	18.18182	21.04072	13.23722
Cont002	16.10942	12.44493	15.06148	18.98831	17.41367	23.49453	23.60877	15.77554	25.42976	17.99894	16.08133	19.11869	18.90694
Cont003	21.02088	23.11828	19.03007	21.46751	21.63501	21.42516	23.98903	15.55137	20.56452	25.57545	23.13167	24.02827	15.09434
Cont004	20.71087	16.81804	14.83845	18.38978	14.85986	30.17975	28.86506	28.47779	22.35741	16.56953	17.60644	17.29107	19.83193
Cont005	15.30612	25.17971	21.71212	19.24645	12.91416	14.9411	26.87802	18.85645	15.76087	16.3031	15.43287	18.86477	9.913259
Cont006	7.836457	13.48089	10.1844	14.13825	18.83734	24	18.13765	18.98513	17.56272	17.98658	15.32399	16.37744	24.13019
Cont007	13.10044	10.71361	12.15794	19.16648	21.15474	25.78861	31.43657	18.93134	25.29142	17.54411	25.19579	25.76387	19.8123
Cont008	21.6285	18.62197	15.63518	21.80747	26.33073	17.84141	20.52402	16.92308	19.81506	17.05539	13.39915	15.61644	19.66527
Cont009	26.78571	20.44374	20.1382	21.49733	19.98167	15.74697	17.92829	16.32653	13.93678	20.42254	18.48014	12.07921	13.66224
Cont010	16.21622	23.89135	11.16337	17.40772	19.17192	26.08696	19.72371	21.51102	21.56698	26.26263	18.51852	22.86501	19.48882
Cont011	14.43515	10.28417	19.28808	23.96333	25.32663	22.43902	27.03102	18.15287	19.71154	20.83333	17.53681	18.95113	14.8802
Mean	17.21771	18.05746	16.17517	19.40768	20.28881	22.30083	24.20945	18.90271	20.4819	19.56269	18.08078	19.27242	17.14752
Standard deviation	4.835495	5.358399	3.706655	2.561401	4.130966	4.388141	4.423095	3.448833	3.502782	3.29084	3.268915	3.795917	3.897664

Higher frequency band (81–500 Hz).

Knee OA Group	35–40%	40–45%	45–50%	50–55%	55–60%	60–65%	65–70%	70–75%	75–80%	80–85%	85–90%	90–95%	95–100%
OA001	24.58472	20.72785	30.61041	38.74539	37.14586	53.99423	52.79018	50.25974	60.07463	67.73455	56.62651	57.96178	56.96203
OA002	26.15327	27.81418	28.15343	44.74429	54.64733	63.50806	60.49166	60.69915	72.0121	70	68.92361	65.33333	67.77042
OA003	9.975369	13.65777	16.71175	23.83815	33.29634	49.03934	49.20914	50.82267	57.47573	58.6821	63.76537	66.00442	57.13058
OA004	13.50267	27.06186	24.24723	22.41224	27.01299	31.79104	34.45826	43.97706	53.01508	53.06748	55.67847	60.4	48.93455
OA005	15.70681	20.2697	24.93359	49.05458	53.33333	59.44842	55.50964	62.0506	52.75735	62.55924	77.34657	70.91413	64.77419
OA006	26.19048	26.71329	27.51951	39.92229	52.68692	58.21727	65.56391	66.26298	73.21429	69.04762	68.28358	74.05213	61.6092
OA007	24.5	23.19328	25.7248	23.73444	40.76016	36.82678	50.9901	45.02618	47.56757	46.3357	59.86842	55.57404	56.60661
OA008	29.6355	26.96035	34.3107	47.01436	52.51018	52.57634	58.70787	55.84615	58.85417	64.03873	65.02463	64.92754	66.72535
OA009	16.82379	21.95467	19.08184	36.42659	50.68182	45.24076	45.98765	51.5102	58.06452	55.15409	53.72727	64.57229	59.86278
OA010	44.07827	52.46407	61.92843	59.51265	73.0102	62.13235	75.3663	65.59679	80.0227	75.67901	82.4165	83.28817	75.20891
OA011	30.53671	18.77117	27.53559	44.48956	38.26745	40.28838	32.98969	37.2549	43.03097	46.01542	46.13139	54.10095	52.18121
OA012	19.93499	23.6712	32.0017	38.76024	35.47352	44.66368	51.94599	50.57712	58.62458	56.58492	54.32756	60.80692	46.36511
OA013	15.352829	20.963099	18.23078	38.764233	41.953385	43.607706	38.573086	47.112462	55.141658	49.565217	58.184143	55.799373	55.694444
Mean	22.84426	24.94019	28.53767	39.03223	45.44457	49.33341	51.73719	52.84585	59.21964	59.57416	62.33108	64.13347	59.21734
Standard deviation	8.721078	8.822391	10.88952	10.3587	11.69427	9.590055	11.61384	8.46474	9.97146	9.132049	9.678161	7.95898	7.702959

Control group	35–40%	40–45%	45–50%	50–55%	55–60%	60–65%	65–70%	70–75%	75–80%	80–85%	85–90%	90–95%	95–100%
Cont001	14.78029	20.72072	17.45738	34.37664	33.1604	35.47215	42.54545	46.43338	54.86726	60.9319	55.8042	59.04977	68.28309
Cont002	14.58967	16.29956	29.5082	43.34011	46.17928	46.39599	51.93929	40.02118	39.18198	55.79672	50.04621	50.24876	47.04579
Cont003	12.34339	23.46041	17.74976	31.92872	43.18745	45.57628	46.94997	56.17342	47.17742	60.35806	54.4484	55.12367	62.26415
Cont004	26.76008	17.55402	27.80215	41.80036	51.13696	31.69347	42.71671	43.11726	51.17871	52.37302	61.44994	59.94236	52.35294
Cont005	16.69985	19.05936	21.31515	40.93949	65.21084	56.97458	49.55203	56.44769	63.17935	61.76808	63.48808	55.92654	68.40149
Cont006	10.39182	10.81489	16.56738	33.53144	37.89827	25.5873	46.23482	47.50656	46.23656	51.00671	45.00876	50.21692	48.48485
Cont007	6.877729	10.61947	7.779515	27.97115	29.31864	32.89635	34.34391	38.98305	46.06418	43.58281	41.65361	49.80718	48.11001
Cont008	24.17303	21.04283	33.17047	37.13163	49.53868	58.70044	48.03493	63.4965	61.95509	71.2828	70.94499	69.72603	67.15481
Cont009	22.02381	25.51506	26.45607	41.71123	53.98717	55.58546	59.76096	59.32945	63.50575	60.73944	63.21244	74.25743	65.65465
Cont010	11.71171	15.63193	20.69614	34.73154	46.17462	44.66403	50.88258	43.75656	55.09688	48.05195	53.59477	53.16804	61.5016
Cont011	11.50628	18.33559	21.10927	35.22479	35.47739	32.35772	35.1551	55.30786	53.24519	56.06061	46.85408	55.42312	60.40353
Mean	15.62342	18.0958	21.78286	36.60792	44.66088	42.35489	46.19234	50.05208	52.88076	56.5411	55.13686	57.53544	59.05972
Standard deviation	5.938257	4.460772	6.792732	4.609578	9.906362	10.95278	7.019308	7.995807	7.531616	7.291861	8.564741	7.595725	8.092866
